# From molecular promise to preclinical results: HDAC inhibitors in the race for healthy aging drugs

**DOI:** 10.15252/emmm.201809854

**Published:** 2019-08-01

**Authors:** Rebecca L McIntyre, Eileen G Daniels, Marte Molenaars, Riekelt H Houtkooper, Georges E Janssens

**Affiliations:** ^1^ Laboratory Genetic Metabolic Diseases Amsterdam UMC Amsterdam Gastroenterology and Metabolism Amsterdam Cardiovascular Sciences University of Amsterdam Amsterdam The Netherlands

**Keywords:** epigenetics, geroprotector, hallmarks of aging, HDAC inhibitors, preclinical models, Ageing, Chemical Biology

## Abstract

Reversing or slowing the aging process brings great promise to treat or prevent age‐related disease, and targeting the hallmarks of aging is a strategy to achieve this. Epigenetics affects several if not all of the hallmarks of aging and has therefore emerged as a central target for intervention. One component of epigenetic regulation involves histone deacetylases (HDAC), which include the “classical” histone deacetylases (of class I, II, and IV) and sirtuin deacetylases (of class III). While targeting sirtuins for healthy aging has been extensively reviewed elsewhere, this review focuses on pharmacologically inhibiting the classical HDACs to promote health and longevity. We describe the theories of how classical HDAC inhibitors may operate to increase lifespan, supported by studies in model organisms. Furthermore, we explore potential mechanisms of how HDAC inhibitors may have such a strong grasp on health and longevity, summarizing their links to other hallmarks of aging. Finally, we show the wide range of age‐related preclinical disease models, ranging from neurodegeneration to heart disease, diabetes to sarcopenia, which show improvement upon HDAC inhibition.

**Glossary**
Adaptive thermogenesis: The regulated production of heat in response to environmental changes in temperature and diet.
AMPK: 5′ AMP‐activated protein kinase is a signaling protein that helps control cellular energy homeostasis.
Ataxia: A neurological sign consisting of lack of voluntary coordination of muscle movements that can include gait abnormality, speech changes, and abnormalities in eye movements.
Cockayne syndrome (CS): A rare disease that is marked especially by growth and developmental failure, photosensitivity, and premature aging.
Embryonic stem cells or ESCs: Stem cells derived from the undifferentiated inner mass cells of a human embryo.
Epigenetics: Heritable changes to phenotype that do not alter DNA sequence including alterations in DNA methylation patterns, post‐translational modification of histones, and chromatin remodeling.
Genotoxic: Damaging to the genetic information within a cell, causing mutations that may lead to cancer.
Geroprotective: Influencing a pathway or aspect of the aging process, thereby prolonging age or intervening in age‐related disease.
Hormesis: A process in which exposure to a low dose of a chemical agent or environmental factor that is damaging at higher doses induces an adaptive beneficial effect on the cell or organism.
Hutchinson–Gilford progeria syndrome (HGPS): Extremely rare autosomal dominant genetic disorder in which symptoms resembling aspects of aging are manifested at a very early age.
IGF1: Insulin‐like growth factor is a hormone that plays a critical role in growth during development and has anabolic effects in adults.
Inflammaging: Low‐grade chronic systemic inflammation established during physiological aging.
Interleukins: Any of a class of glycoproteins produced by leukocytes for regulating immune responses.
mTOR: Mechanistic target of rapamycin is a signaling protein that helps control cellular division and survival.
NF‐κB: Protein complex that controls transcription of DNA, cytokine production, and cell survival.
Sarcopenia: Degenerative loss of skeletal muscle mass and strength with aging.
Senescence: The condition or process of deterioration with age. Cellular senescence describes the loss of a cell's ability to grow or divide.
Spatial memory: Cognitive function that allows us for the recall of three‐dimensional objects or places.
Steatosis: Accumulation of fat in liver cells, associated with disturbance of the metabolism.
Synovium: A membrane that lines a joint or surrounds a tendon and releases fluid allowing for joint movement.
Xeroderma pigmentosum: A rare hereditary defect of the enzyme system that repairs DNA after damage from ultraviolet rays, resulting in extreme sensitivity to sunlight and a tendency to develop skin cancer.

## Introduction

It has become increasingly clear that epigenetics, including DNA methylation, histone modifications, and chromatin state, play a crucial role in the aging process (López‐Otín *et al*, 2013). For example, by assessing changes in DNA methylation patterns, a person's age can be predicted within 5 years of accuracy (Field *et al*, 2018). Histone modifications, including methylation and acetylation states, have been intimately linked to lifespan regulation (Maleszewska *et al*, 2016). Together, these modifications dictate chromatin state, affecting both gene transcription and genome stability. Epigenetic changes occurring with age provide a tantalizing therapeutic target. In contrast to DNA mutations, epigenetic alterations represent reversible changes, offering the potential for a true “rejuvenating” therapeutic intervention. Of the various epigenetic alterations occurring with age, the influence of histone acetylation, a process balanced by the activity of histone acetyltransferases (HATs) and histone deacetylases (HDACs), on lifespan regulation has been the most characterized, mainly due to the advent of HDAC inhibitors from the cancer biology field (Li & Seto, 2016).

Genes encoding HDACs are divided into four classes based on their homology to their yeast counterparts (Willis‐Martinez *et al*, 2010). Class I HDACs, which are most similar to the yeast RPD3 gene, include HDACs 1, 2, 3, and 8. Class II HDACs, which are most similar to the yeast HDA1 gene, include HDACs 4, 5, 6, 7, and 9. Class IV includes only one member, HDAC11, which is similar to both RPD3 and HDA1. Classes I, II, and IV are considered to form the “classical family” of HDACs, being dependent on the Zn^2+^ ion for their activity. HDACs of class III, rather, are dependent on NAD^+^ and comprise the sirtuin family of proteins. The role of sirtuin‐based pharmacological intervention in aging has been covered previously and will therefore not be part of this review (Houtkooper *et al*, 2012; Bonkowski & Sinclair, 2016). Likewise, reviewing the relation of all epigenetic mechanisms to healthy aging is beyond the scope of this work. The focus of this review will be limited to the classical HDACs specifically, to recapitulate the many findings related to the beneficial effects on health and aging that result from their inhibition. We aim to provide a broad overview to introduce the reader in the diverse fields involved and to facilitate deeper investigation.

## Mechanisms of lifespan extension resulting from HDAC inhibition

Yeasts share many hallmarks of aging with humans, and specifically, a yeast mutant lacking the histone deacetylase gene RPD3 has a prolonged lifespan (Kim *et al*, 1999; Janssens & Veenhoff, 2016). Interestingly, a yeast strain lacking the HDA1 gene does not show longevity benefits, perhaps implicating class I HDACs above others in the aging process (Kim *et al*, 1999). In worms, three class I HDACs exist, *hda‐1, ‐2,* and *‐3*, of which RNAi knockdown of *hda‐2* and *‐3* increases lifespan (Edwards *et al*, 2014). This is in line with the observation that valproic acid and β‐hydroxybutyrate (BHB), both class I selective HDAC inhibitors, also increase worm lifespan (Evason *et al*, 2008; Edwards *et al*, 2014). Indeed, lifespan extension by BHB in worms depends on the HDAC genes (Edwards *et al*, 2014). While genetic lifespan studies with HDACs have been mainly performed in yeast and worms, research in flies has arguably contributed the most to our understanding of HDAC inhibitors as longevity drugs (Table 1 and Pasyukova & Vaiserman, 2017). Fly lifespan increased upon treatment with the class I and II HDAC inhibitors phenylbutyrate (Kang *et al*, 2002), and sodium butyrate (Zhao, 2005), both of which are types of short‐chain fatty acids. Trichostatin A, a hydroxamic acid targeting classes I, II, and IV HDACs, also increased fly lifespan (Tao *et al*, 2004), as does vorinostat (also known as SAHA), another hydroxamic acid (McDonald *et al*, 2013).

**Table 1:** Properties of selected HDAC inhibitors

HDACi	HDAC1 IC_50_ (μM)	HDAC class specificity	Structural class	Lifespan extension
Valproic acid	171	I	Short‐chain fatty acid	Worms (Evason *et al*, 2008)
Phenylbutyrate	162	I, II	Short‐chain fatty acid	Flies (Kang *et al*, 2002)
Butyrate	175	I, II	Short‐chain fatty acid	Flies (Zhao, 2005)
β‐hydroxybutyrate (BHB)	5,300	I	Ketone body	Worms (Edwards *et al*, 2014)
Trichostatin A	0.017	I, II, IV	Hydroxamic acid	Flies (Tao *et al*, 2004)
Vorinostat (SAHA)	0.014	I, II, IV	Hydroxamic acid	Flies (McDonald *et al*, 2013)
Scriptaid	0.0064	I	Hydroxamic acid	NA
Apicidin	0.00030	I	Cyclic peptide	NA
MS‐275 (Entinostat)	0.5	I	Benzamide	NA
Merck60	0.007	I	Benzamide	NA

HDAC inhibitors are as described in the text. All HDAC inhibitors listed here target at least class I HDACs, and therefore, the IC50 of HDAC1 is given as reference (though certain HDACs may have slightly higher or lower specificities to other HDACs within class I; Hu, 2003; Huber *et al*, 2011; Shimazu *et al*, 2013; Frys *et al*, 2015). General HDAC class inhibition is listed for added consideration (Shimazu *et al*, 2013; Stubbs *et al*, 2015; Pasyukova & Vaiserman, 2017). While all compounds inhibit HDACs, several, including the short‐chain fatty acids and ketone body, have other roles in the cell (Shimazu *et al*, 2013; Stubbs *et al*, 2015; Pasyukova & Vaiserman, 2017). The majority listed has been shown to increase lifespan in at least one model organism.

**Figure 1:**
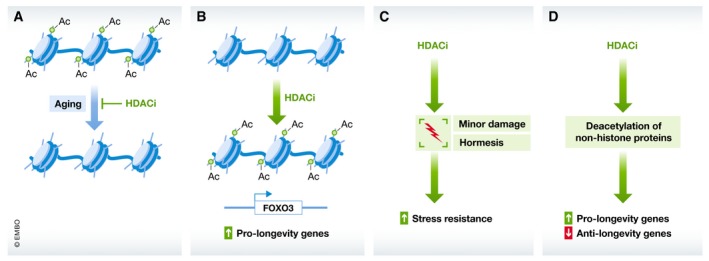
Potential models whereby HDAC inhibition (HDACi) extends lifespan (A) HDACi may directly reverse age‐related deacetylation of chromatin, reverting the epigenome back to a more youthful state. (B) HDACi may result in acetylation of histones near pro‐longevity genes, increasing their transcription. (C) HDACi may act through a hormesis effect, causing low dose damage that activates stress resistance, resulting in a net benefit for the organism. (D) HDACi may target non‐histone proteins, activating pro‐longevity proteins, and/or de‐activating anti‐longevity proteins.

The exact means by which HDAC inhibitors extend lifespan has not been fully resolved; however, a number of possible mechanisms can be envisioned (Fig 1). One possible scenario is that HDAC inhibitors reverse the natural age‐related changes occurring in the histone acetylation landscape (Fig 1A). This is the most simple explanation for their benefits, supported by the observation that many acetylation marks on histones generally decrease with age and in certain age‐related diseases (Peleg *et al*, 2016). A second possible mechanism of HDAC inhibitors is that they may affect histones and nucleosomes to directly activate transcription of pro‐longevity genes (Fig 1B). This is supported by observations that an endogenous HDAC inhibitor, β‐hydroxybutyrate (BHB), can increase acetylation in the promoter of the pro‐longevity transcription factor FOXO3a resulting in its increased expression, and indeed, BHB's lifespan extending effects depend on HDAC genes (Shimazu *et al*, 2013; Edwards *et al*, 2014). A third possible mechanism through which HDAC inhibitors may increase lifespan is through hormesis (Fig 1C). In this scenario, while high doses of HDAC inhibitors may be toxic, low doses would elicit activation of protective genes to regain homeostasis, ultimately improving function (Vaiserman, 2011). This is supported by observations that flies treated with HDAC inhibitors show upregulation of heat shock protein chaperones, a class of genes that are usually upregulated under stress (Zhao, 2005). A fourth possibility is that HDAC inhibitors may regulate lifespan by modifying the acetylation state of non‐histone proteins, activating signaling cascades that promote longevity independent of histone modifications (Lu *et al*, 2011; Singh *et al*, 2010; Fig 1D).

The most likely scenario is that HDAC inhibitors act through combinations of these mechanisms, dependent on the dose, cell type, and drug involved in the experiment. While the mechanism may not yet be fully resolved, their benefit to the aging process at molecular and preclinical levels is clear.

## HDAC inhibitors and the hallmarks of aging

**Figure 2:**
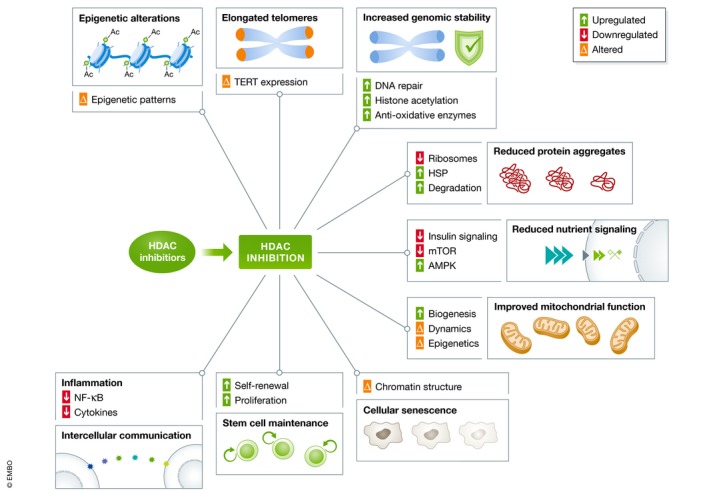
Influence of HDAC inhibition on the hallmarks of aging HDAC inhibitors are listed as described in the text, along with evidence for the benefits they impart at the molecular level on the hallmarks of aging; epigenetic alterations, telomere attrition, genomic instability, loss of proteostasis, deregulated nutrient sensing, mitochondrial dysfunction, cellular senescence, stem cell exhaustion, and altered intercellular communication, and up‐ or downregulated processes are generally in line with beneficial changes for the health of the organism, while altered changes are indicative of potential synergies and interactions.

The hallmarks of aging are molecular changes associated with aging that are likely to cause the aging process and, when inhibited, should slow the onset of aging (López‐Otín *et al*, 2013). Nine hallmarks have been established to date (Fig 2), and these are sub‐divided into primary hallmarks (those responsible for age‐related cellular damage), antagonistic hallmarks (those that act as responses to damage), and integrative hallmarks (those that are considered culprits of age‐related phenotypes). While some hallmarks have been greatly ameliorated by treatment with HDAC inhibitors, others are only now emerging with potential links. Below, we list evidence of how HDAC inhibitors can directly and indirectly influence the nine hallmarks of aging (Fig 2).

### Primary hallmarks

#### Epigenetic alterations

The clearest link between the hallmarks of aging and HDAC inhibitors exists with the hallmark of “epigenetic alterations” since histone deacetylases are one of the several types of enzymes able to induce alterations in epigenetic patterns (Hirst & Marra, 2013). Many acetylation markers on histones decrease with age, including bulk histone 4 acetylation levels and histone 3 acetylation at lysine residues 18, 27, and 56, which is thought to facilitate the aging process (Feser & Tyler, 2011). Therefore, the effects of an HDAC inhibitor, which prevents HDACs from removing acetyl groups further, have the potential to directly reverse or prevent these age‐related changes. The role of epigenetic alterations in aging can be interconnected with other hallmarks, broadening the reach HDAC inhibitors have to positively benefit aging at the molecular level.

#### Telomere attrition

Telomeres are protective repetitive sequences located at the ends of chromosomes. These sequences can only be completely replicated by telomerase, and more specifically, the catalytic subunit telomerase reverse transcriptase (TERT), which is transcriptionally repressed in the majority of adult somatic cells (Shay & Wright, 2011). Telomere shortening has been observed during normal aging in both humans and rodents, and using animal models, a causal link has been established between telomere loss and organismal aging (López‐Otín *et al*, 2013).

Upon discovery that histone deacetylation is essential to the transcriptional regulation of the *TERT* gene (Cong & Bacchetti, 2000; Hou *et al*, 2002), early research attempted to elucidate the impact on telomeres. However, these resulting studies conflict in their findings. Even within the same cell types, C33A cancer cells, one study finds increased TERT expression (Takakura *et al*, 2001) with TSA treatment, while another finds no effect (Hou *et al*, 2002). In a liver cancer cell line, TSA was even shown to reduce telomerase activity (Nakamura *et al*, 2001). More recently, inhibition of histone deacetylation during vascular remodeling using the HDAC inhibitor scriptaid led to activation of *TERT* transcription but decreased *TERT* protein abundance (Qing *et al*, 2016). Telomere lengthening was shown in mouse embryonic stem cells upon treatment with sodium butyrate, without a change in *Tert* gene expression (Dan *et al*, 2015). In line with this, trichostatin A can lengthen telomeres in cloned pigs during somatic cell nuclear transfer (Kong *et al*, 2014). Taken together, these studies suggest that HDAC inhibitors may have distinct effects on the TERT gene depending on experimental conditions and the treated cell type. Importantly though, HDAC inhibition is connected to telomere lengthening, the main outcome necessary to reverse the hallmark of aging of telomere attrition.

#### Genomic instability

The accumulation of genetic damage such as mutations contributes to the aging process (López‐Otín *et al*, 2013). To maintain genomic integrity and stability, DNA repair mechanisms are present in the cell to restore these lesions. Since HDAC inhibitors originate from the cancer biology field, and because causing damage to DNA and impairing DNA lesion repair may be a desirable trait to reduce proliferation of cancerous cells, HDAC inhibitors have been studied for their ability to accelerate DNA damage and reduce DNA repair (Robert & Rassool, 2012).

The dual role of HDAC inhibitors in cancer versus normal cells is clear. While irreparable DNA damage occurs upon treatment with vorinostat in transformed cells, normal cells do not seem to suffer from this effect (Lee *et al*, 2010). This may be due to the fact that HDACs are generally overexpressed in cancerous cells compared to normal cells (Roos & Krumm, 2016), which may allow for a titrated dose to impart selectively beneficial effects. When studied in a non‐cancerous setting, sodium butyrate treatment stimulated DNA repair after UV‐irradiation in human fibroblasts (Smerdon *et al*, 1982). This was also the case in xeroderma pigmentosum fibroblasts, a cell type especially prone to DNA damage (Smerdon *et al*, 1982). Sodium butyrate also reduced hydrogen peroxide‐induced DNA damage in rat bone marrow cells, further revealing the potential for this HDAC inhibitor's anti‐genotoxic effects (El‐Shorbagy, 2017). Taken together, HDAC inhibitors may provide multiple routes to ensuring genomic stability, from boosting repair capacity in normal cells to promoting detoxification of potential DNA damaging agents.

#### Loss of proteostasis

The cellular homeostasis of proteins involves (i) their biogenesis by ribosomes, (ii) their folding by chaperones, and (iii) their degradation by proteasomes and autophagy. Inhibition of HDACs may benefit aging at all three steps of proteostasis. For instance, the first step to facilitate biogenesis of ribosomes is transcription of ribosomal DNA. HDAC1 modulates ribosomal DNA transcription, as HDAC1‐overexpressing cells revealed an increase in total ribosomal RNA (Meraner *et al*, 2008). Moreover, double treatment with the HDAC inhibitor trichostatin A and an mTOR inhibitor synergistically reduced polyribosome formation (Wilson‐Edell *et al*, 2014). Long‐lived model organisms are often marked by reduced polyribosome formation (Stout *et al*, 2013; Molenaars *et al*, 2018), suggesting HDAC inhibitors may act similarly through this pathway.

Once proteins are synthesized by ribosomes, multiple quality control mechanisms ensure their stability and functionality, including protein chaperones such as the heat shock proteins (HSPs). The heat shock response plays a beneficial role in lifespan regulation (Hsu *et al*, 2003). In relation to this, treatment with HDAC inhibitors in *Drosophila* resulted in altered chromatin morphology at HSP gene loci and elevated HSP expression, accompanying lifespan extension (Tao *et al*, 2004; Zhao, 2005). This suggests HDAC inhibitors may contribute to lifespan extension also by protein quality assurance pathways.

Finally, the last step in proteostasis involves the decomposition of proteins, performed either by proteasomal degradation or autophagy, both of which play key roles in aging and can be regulated by HDACs (Scognamiglio *et al*, 2008; Trüe & Matthias, 2012; Milota *et al*, 2013; Cellerino & Ori, 2017; Kong *et al*, 2017). For instance, HDAC inhibitor treatments in several cancer cell lines activate the ubiquitin–proteasome pathway, leading to increased protein degradation (Scognamiglio *et al*, 2008; Hakami *et al*, 2016; Kong *et al*, 2017). HDAC inhibitors also induce autophagy (Hrzenjak *et al*, 2008; Liu *et al*, 2010), as does genetic knockdown of HDAC1 (Oh *et al*, 2008). Taken together, these findings suggest that HDAC inhibition provides benefits at all steps required for proteostasis, and directly act to ameliorate this hallmark of aging.

### Antagonistic hallmarks

#### Deregulated nutrient signaling

Nutrient signaling pathways, such as insulin/IGF1, mTOR, sirtuins, and AMPK, transmit cellular signals about nutrient availability to regulate the processes of growth and autophagy. Strong evidence suggests that increased growth signaling through mTOR or IGF1 accelerates aging, while their inhibition or downregulation, for example, via sirtuin or AMPK activation, extends lifespan (Houtkooper *et al*, 2010). Similarly, one of the most widely demonstrated lifespan lengthening interventions, calorie restriction, imparts its benefits through nutrient sensing pathways.

Calorie restriction, a treatment that increases the abundance of the endogenous HDAC inhibitor and ketone body β‐hydroxybutyrate (BHB), or administration of BHB alone, similarly increased global histone acetylation in mouse tissues and protected cells from oxidative stress and damage (Shimazu *et al*, 2013). Butyrate also increased phosphorylation of AMPK in both the liver and muscle of mice (Gao *et al*, 2009), suggesting its activation of metabolic longevity networks. Furthermore, the HDAC inhibitors apicidin and trichostatin A reduced mTOR activation (Morales *et al*, 2016), while an independent study showed trichostatin A and vorinostat downregulate insulin signaling (Kawada *et al*, 2017). This is supported by the observation that vorinostat treatment could reduce phosphorylation of the insulin receptor β (Dudakovic *et al*, 2013). Taken together, these studies show HDAC inhibitors can module nutrient signaling pathways in a manner beneficial to the aging process.

#### Mitochondrial dysfunction

With age, a number of mitochondrial regulatory factors diminish, leading to mitochondria with a decreased capacity for energy generation, as well as increased accumulation of damage and reduced mitochondrial turnover. These factors can include mutations in mtDNA, oxidation of mitochondrial proteins, destabilization of respiratory chain complexes, changes in composition of the mitochondrial membrane, alterations in dynamics, and insufficient quality control by mitophagy (López‐Otín *et al*, 2013). Intervening in mitochondrial biology can increase lifespan (Andreux *et al*, 2013; Houtkooper *et al*, 2013; Sun *et al*, 2016).

There is strong evidence that HDAC inhibitors can prevent or reverse some of this deterioration. Butyrate has been demonstrated in several studies to elevate mitochondrial biogenesis, leading to increases in oxygen consumption (Gao *et al*, 2009; Galmozzi *et al*, 2013; Walsh *et al*, 2015). Additionally, a variety of HDAC inhibitors lead to mitochondrial elongation by creating an imbalance in mitochondrial fission and fusion proteins, demonstrating the influence of HDACs on mitochondrial biology (Lee *et al*, 2012). Despite the lack of histones in mitochondrial DNA (Rebelo *et al*, 2011), HDAC inhibitors can also alter mtDNA epigenetics. Under long exposure to valproic acid or MS‐275, mtDNA methylation was significantly decreased, potentially due to a nuclear deacetylase inhibition of TET (ten‐eleven translocation) enzymes (Chen *et al*, 2012). These findings provide an interesting connection between nuclear and mitochondrial epigenetics and their potential influence on one another, and link HDAC inhibition to desirable effects for mitochondria during aging.

#### Cellular senescence

Inducing senescence—a quiescent, non‐dividing cell state—in cancerous cells is a desired outcome for cancer therapy, and a number of studies have shown HDAC inhibitors to cause various cancer cells to senesce (Lorenz *et al*, 2011; Vargas *et al*, 2014; Venkatesh *et al*, 2015). In the context of aging however, senescent cell *reduction*, rather than *promotion*, is desired, since senescent cells contribute to age‐related diseases (De Keizer, 2017). Importantly, the ability of HDAC inhibitors to cause cell senescence may be cancer specific; sodium butyrate was shown to potentiate senescence in human and rat glioma cell lines but not in normal astrocytes (Vargas *et al*, 2014). Furthermore, as the senescent cell state is reinforced by the chromatin state at the epigenetic level (Narita *et al*, 2003; Funayama & Ishikawa, 2007), there is high potential for HDAC inhibitor involvement in the phenotype. However, the potential benefit HDAC inhibitors may provide to ameliorate the hallmark of cellular senescence is a relatively unexplored field. Overexpression of HDAC1 in melanocytes induced epigenetic pathways leading to growth arrest and senescence (Bandyopadhyay *et al*, 2007), suggesting that a general strategy of HDAC inhibition may limit the tendency of cell senescence to occur. Indeed, endogenously high levels of HDAC1 are present in certain senescent cells, implying a hyperactivity for which inhibition may also be beneficial (Soliman *et al*, 2008). While HDAC1 has been highly implicated in senescence and disease, more studies are required to link other HDACs and potential benefits of HDAC inhibitors to cellular senescence (reviewed in Willis‐Martinez *et al*, 2010).

### Integrative hallmarks

#### Stem cell exhaustion

The loss of the regenerative capacity of tissues is another well‐known characteristic of the aging process (López‐Otín *et al*, 2013). Upon aging, the loss of quiescence and self‐renewal capacity occurs in several types of stem cells (Chakkalakal *et al*, 2012). A potential mechanism for stem cell fate is the epigenetic modification of chromatin, such as histone acetylation state, and the chromatin status of pluripotency genes is believed to be important for stem cell identity and self‐renewal (Bibikova *et al*, 2008). This places drugs that modify chromatin state, such as HDAC inhibitors, as potential regulators of stem cells in aging.

Treatment of embryonic stem cells (ESCs) with trichostatin A or sodium butyrate increased their resistance to oxidant stresses, thereby promoting cell viability and decreasing apoptosis in this primal stem cell population (Chen *et al*, 2011). This may explain an earlier report demonstrating that treatment of ESCs with butyrate activates a self‐renewal program and that HDAC inhibitors generally support human ESC self‐renewal (Ware *et al*, 2009). Adding low concentrations of the HDAC inhibitors largazole or trichostatin A promoted mesenchymal stem cell proliferation, suppressing differentiation and thereby maintaining the self‐renewal capacity of the stem cells (Wang *et al*, 2013). Trichostatin A also upregulated several cyclins, promoting proliferative capacity in adult stem cells (Dhoke *et al*, 2016). Taken together, these studies suggest that HDAC inhibitors have an ability to combat the aging hallmark of stem cell exhaustion.

#### Altered intercellular communication

One of the main changes in intercellular communication occurring during aging is an increase in the pro‐inflammatory phenotype known as “inflammaging” (López‐Otín *et al*, 2013). This state is characterized by an over‐activation of the NF‐κB pathway and increases in pro‐inflammatory cytokines including interferon gamma (IFN‐gamma) and interleukins such as IL‐6 and IL‐12. This results in increased inflammation throughout the body, pro‐inflammatory tissue damage, and a reduced efficacy of the immune system (Franceschi *et al*, 2000, 2018). Inflammaging has been linked to many age‐related diseases including atherosclerosis, heart disease, type II diabetes, arthritis, neurodegeneration, and cancer, suggesting anti‐inflammatory agents could benefit aging and age‐related disease (Xia *et al*, 2016).

HDAC inhibitors have anti‐inflammatory properties, as reviewed in (Adcock, 2007). Activity of HDAC1 correlates to inflammation markers in patients with rheumatoid arthritis (Horiuchi *et al*, 2009). In line with this, the HDAC inhibitor MS‐275, which inhibits HDAC1 and 3, had anti‐arthritic activity and resulted in anti‐inflammatory effects in mice and rats (Lin *et al*, 2007). Phenylbutyrate and trichostatin A also reduced expression of tumor necrosis factor‐alpha (TNF‐alfa) in an animal model of rheumatoid arthritis (Chung *et al*, 2003). In line with this, a single treatment with vorinostat in mice was able to reduce circulating TNF‐alfa, IL‐1‐beta, IL‐6, and IFN‐gamma levels induced by the inflammation inducer lipopolysaccharide (Leoni *et al*, 2002). Furthermore, butyrate was also able to reduce NF‐κB activity in macrophages of Crohn's disease patients (Lührs *et al*, 2002). Taken together, these studies show the ability of HDAC inhibitors to suppress inflammatory signals, which are upregulated during aging and constitute a majority of age‐related altered intercellular communication.

## Benefits of HDAC inhibitors in preclinical age‐related disease models

**Figure 3:**
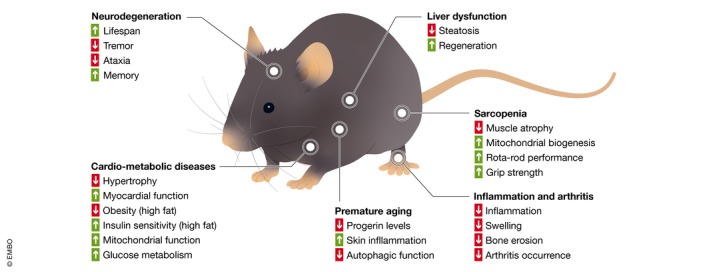
Benefits of HDAC inhibitors in preclinical models HDAC inhibition benefits a large variety of preclinical models, including those related to neurodegeneration, cardio‐metabolic deficiencies, liver dysfunctions, sarcopenia, inflammation‐related disease, and diseases of premature aging. Specifics of improvements in each condition are described in the text.

HDAC inhibitors have shown benefits in diverse preclinical models for age‐related diseases, likely owing to their broad effects at the molecular level as described above. Here, we review evidence that HDAC inhibitors can provide either prophylactic treatment for disease reduction, or treatment after onset, for a variety of age‐related diseases. With cancer considerations aside, most evidence to date for the application of HDAC inhibitors exists for neurodegeneration, cardio‐metabolic diseases, liver dysfunction, sarcopenia, inflammation and arthritis, and diseases of premature aging in preclinical mouse studies (Fig 3). While evidence for HDAC inhibitors as treatments is only now emerging for some of these age‐related diseases (such as liver dysfunction or sarcopenia), others have large amounts of evidence and clinical trials are underway (such as for neurodegeneration).

### Neurodegeneration

Perhaps the most convincing case for the non‐cancer use of HDAC inhibitors can be made for treating neurodegenerative diseases, including Alzheimer's and Parkinson's. The effect on HDAC inhibitors on brain function is well known (Fischer *et al*, 2010) and is exemplified by the observation that treatment with the HDAC class I selective inhibitor Merck60 can alter behavior in anxiety mood tests in mice (Lewis *et al*, 2014). In relation to the human aging brain, histone acetylation levels are known to decline (Tang *et al*, 2011), suggesting HDAC inhibition may provide benefit to normal aging too. Histone acetylation levels also play a key role in neurodegenerative diseases (Gangisetty, 2018). Treatment with sodium butyrate extended mean lifespan of Atro‐118Q mice (possessing neuronal expression of a mutant human Atrophin‐1 protein containing an expanded stretch of 118 glutamines) and improved their neurodegenerative phenotypes (tremor, ataxia, and other motor defects; Ying *et al*, 2006). Furthermore, treatment of Alzheimer's disease transgenic mice (dual transgenic expressing mutant forms of both App and Psen1 genes) with phenylbutyrate resulted in a reduction of amyloid plaques in the cortex and hippocampus (Wiley *et al*, 2011). Phenylbutyrate also restored brain histone acetylation levels and reduced Tau pathology in another Alzheimer's disease transgenic mouse model (expressing mutant form of App gene; Ricobaraza *et al*, 2009). In line with this, treatment with the pan‐HDAC inhibitor vorinostat lead to restoration of spatial memory of another Alzheimer's disease mouse model (dual transgenic expressing mutant forms of both App and Psen1; Benito *et al*, 2015). Together, these findings provided justification for a human clinical trial using phenylbutyrate to treat Parkinson's disease (NCT02046434).

### Cardio‐metabolic diseases

Cardio‐metabolic diseases, affecting the heart and metabolic state, such as heart disease, diabetes, and obesity, have age‐related dependencies for their occurrences and outcomes. HDAC inhibition has been shown to provide benefit to the cardio‐metabolic system in mice. Treatment with trichostatin A can reduce pressure overload‐induced cardiac hypertrophy, and results in histone acetylation of genes related to cardiac contraction, collagen deposition, inflammation, and the extracellular matrix (Ooi *et al*, 2015). Additionally, treatment with sodium butyrate in streptozotocin‐induced diabetic mice improved myocardial function as measured by echocardiography and reduced cardiac hypertrophy as made evident by a reduced heart/tibia ratio (Chen *et al*, 2015). Meanwhile, treatment with sodium butyrate of mice fed a high‐fat diet prevented development of obesity and insulin resistance, most likely due to improved mitochondrial function as made evident by improved adaptive thermogenesis and fatty acid oxidation (Gao *et al*, 2009). Mice on a high‐fat diet treated with sodium butyrate showed improved insulin sensitivity (Henagan *et al*, 2015), as did mice on a high‐fat diet treated with vorinostat (Sharma & Taliyan, 2016). Furthermore, treating naturally aged mice with sodium butyrate improved glucose metabolism as discerned from a glucose tolerance test, suggesting an age‐related benefit as well, beyond diet‐induced dysfunctions (Walsh *et al*, 2015). These diverse studies in mice suggest that the cardio‐metabolic system, which diminishes in function with age, benefits from HDAC inhibition.

### Liver dysfunction

HDAC1 plays a crucial role in liver aging and disease with strong links emerging in regard to its role in liver dysfunction (Willis‐Martinez *et al*, 2010). HDAC1 is thought to inhibit liver regeneration in aged mice and liver‐specific overexpression of HDAC1 resulted in steatosis, a marker of liver aging (Wang *et al*, 2008). Acetylation of histone H3K9, an HDAC1 target, decreased in livers of old mice, again suggesting hyperactivity of HDAC1 with age (Kawakami *et al*, 2009). Together, these preliminary findings suggest that aging in the liver results in an increased HDAC activity and that HDAC inhibitors act to reverse the epigenome back toward a more youthful state, preventing steatosis and improving regenerative potential.

### Sarcopenia

HDACs play key roles in regulating metabolism in skeletal muscle (Walsh & Van Remmen, 2016). For example, reduction in the level of HDAC1 or inhibition of its activity prevents muscle atrophy after nutrient deprivation (Beharry *et al*, 2014). Treating naturally aged mice with sodium butyrate reduced age‐related muscle atrophy and increased mitochondrial biogenesis (Walsh *et al*, 2015). Furthermore, in SOD1‐G93A mice, a model for oxidative damage in aging, treatment with trichostatin A ameliorated muscle atrophy and improved mouse performance in rotarod and grip strength assays (Yoo & Ko, 2011). These preliminary studies suggest that HDAC inhibition may prevent or reverse the muscle atrophy that accompanies aging.

### Inflammation and arthritis

As described above in the hallmark of “altered cellular communication”, HDAC inhibitors can act as anti‐inflammatory agents, opening up their application to inflammation‐based diseases (Adcock, 2007). HDAC1 is highly expressed in the synovium of arthritis patients, which correlates to inflammation markers (Horiuchi *et al*, 2009). This suggests HDAC inhibition may provide benefit, and indeed, in both mouse and rat collagen‐induced arthritis models, the HDAC inhibitors vorinostat and MS‐275 had prophylactic activity against swelling and reduced bone erosion (Lin *et al*, 2007). MS‐275 was also able to prevent the onset of arthritis, and when treatment occurred after onset, MS‐275 prevented disease progression and joint destruction (Lin *et al*, 2007). Together, these findings point to HDAC inhibition as not only a means to prevent inflammation‐based age‐related disease, but also as a treatment for them.

### Premature aging

Hutchinson–Gilford progeria syndrome (HGPS) is a rare human genetic disease that leads to severe premature aging, caused by mutations in the *LMNA* gene and characterized by an accumulation of a mutated lamin A precursor (progerin), nuclear dysmorphism, and chromatin disorganization (Columbaro *et al*, 2005; Arancio *et al*, 2014). In HGPS cells, dramatic epigenetic alterations have been reported (reviewed by Arancio *et al*, 2014). In both model cell lines and cells from patients with HGPS, valproic acid and TSA lowered progerin levels, which allowed for rescue of heterochromatin organization and reorganization of transcripts (Columbaro *et al*, 2005; Stephens *et al*, 2017).

Another hereditary form of premature aging, Cockayne syndrome (CS), is caused by mutations in five different genes that encode proteins involved in nucleotide excision DNA repair, causing hypersensitivity to UV radiation and loss of subcutaneous fat (Majora *et al*, 2018). Across *CSB*‐deficient human fibroblasts, *Caenorhabditis elegans*, and mice, treatment with SAHA enhanced alpha‐tubulin acetylation and improved autophagic function, and even rescued the skin phenotype observed in mice, suggesting it may provide a therapeutic option for CS (Majora *et al*, 2018). Together, these findings not only suggest that HDAC inhibitors can provide treatment for diseases of premature aging, but also provide further evidence of HDAC inhibitor efficacy as a geroprotective compound.

## Conclusion

Epigenetics is a major regulator of cell fate and function and is clearly implicated in disease biology (Moosavi & Ardekani, 2016). In this review, we specifically focused on one aspect of epigenetic regulation, HDACs, and more specifically, on the pharmacological benefit of their inhibition. In this regard, HDAC inhibitors can directly target and reverse the age‐related changes of a main hallmark of aging—that of epigenetic alterations. Furthermore, as reviewed here, their influence reaches more broadly to all other hallmarks of aging, likely contributing to their ability to increase lifespan in model organisms. Interventions that reverse or slow the aging process bring great promise to promote healthy aging. HDAC inhibitors have demonstrated themselves to fill this role, providing potential treatments for age‐related diseases ranging from neurodegeneration to heart disease, diabetes to sarcopenia. While this review has specifically focused on pharmacological inhibition of HDACs, genetic knockout of HDACs has also revealed important roles for HDACs in disease biology. For example, germline knockout of either HDAC1 or HDAC2 alone results in lethality, and conditional knockout of both has in general been found to be detrimental to a variety of tissues (e.g., neuronal, cardiovascular, liver, etc; Kelly & Cowley, 2013). Therefore, despite the promising outlook of HDAC inhibitors for healthy aging, much work remains to be done to better understand their safety and how to minimize adverse side effects. Owing to their origins in the cancer biology field, many cell‐type and dose‐dependent negative effects of HDAC inhibitors on cell viability have been documented. Careful optimization of dose and drug pharmacokinetics should be made prior to pursuing any strategy in which HDAC inhibitors would be used as a prophylactic drug for healthy aging. More specifically, less‐toxic versions of current drugs may be required. Understanding of the mechanism by which HDAC inhibitors extend lifespan is noticeably limited, and many mechanistic options remain (Fig 1). Deeper study of the specific modes of action of these compounds is necessary prior to their implementation as geroprotective compounds. Finally, while all compounds discussed within this review have been demonstrated to be HDAC inhibitors, it should be noted that the majority that have been shown to produce lifespan extending effects in model organisms may also have effects other than HDAC activity in the cell (such as the short‐chain fatty acids or ketone bodies, Table 1). Therefore, further evaluation of lifespan extending effects of more potent HDAC inhibitors is also warranted. In conclusion, along with words of caution, this review provides strong molecular and preclinical evidence supporting the further development of HDAC inhibitors for the race to develop healthy aging drugs.

## Conflict of interest

The authors declare that they have no conflict of interest.

**Pending issues**

(i) Identification of mechanism or mechanisms by which HDAC inhibitors extend lifespan.
(ii) Understanding safety of HDAC inhibitors *in vivo* and how to minimize potential adverse effects.
(iii) Dose optimization and pharmacokinetic studies of HDAC as geroprotective compounds.
(iv) Development of specifically targeted or less toxic HDAC inhibitor compounds.
